# Muscle Strength, Physical Activity, and Functional Limitations in Older Adults with Central Obesity

**DOI:** 10.1155/2016/8387324

**Published:** 2016-02-29

**Authors:** Cassandra M. Germain, John A. Batsis, Elizabeth Vasquez, Douglas R. McQuoid

**Affiliations:** ^1^Department of Psychiatry and Behavioral Sciences, Duke University Medical Center, P.O. Box 3119, Durham, NC 27710, USA; ^2^Department of Medicine, Geisel School of Medicine at Dartmouth, Dartmouth-Hitchcock Medical Center, 1 Medical Center Drive, Lebanon, NH 03756, USA; ^3^Department of Epidemiology and Biostatistics, School of Public Health, University at Albany (SUNY), One University Place, Albany, NY 12203, USA

## Abstract

*Background.* Obesity and muscle weakness are independently associated with increased risk of physical and functional impairment in older adults. It is unknown whether physical activity (PA) and muscle strength combined provide added protection against functional impairment. This study examines the association between muscle strength, PA, and functional outcomes in older adults with central obesity.* Methods.* Prevalence and odds of physical (PL), ADL, and IADL limitation were calculated for 6,388 community dwelling adults aged ≥ 60 with central obesity. Individuals were stratified by sex-specific hand grip tertiles and PA. Logistic models were adjusted for age, education, comorbidities, and body-mass index and weighted.* Results.* Overall prevalence of PL and ADL and IADL limitations were progressively lower by grip category. Within grip categories, prevalence was lower for individuals who were active than those who were inactive. Adjusted models showed significantly lower odds of PL OR 0.42 [0.31, 0.56]; ADL OR 0.60 [0.43, 0.84], and IADL OR 0.46 [0.35, 0.61] for those in the highest grip strength category as compared to those in the lowest grip category.* Conclusion.* Improving grip strength in obese elders who are not able to engage in traditional exercise is important for reducing odds of physical and functional impairment.

## 1. Introduction

Among those aged 60 and older, the combined prevalence of overweight and obesity, as assessed by body mass index (BMI), is estimated at over 70% [1]. The prevalence of central obesity is also rising among older adults in the US [2, 3] and is independently associated with functional impairment and disability [4, 5]. Central obesity is also associated with muscle weakness [6, 7], a known independent risk factor for physical disability [8] and mortality in older adults [9]. Thus, adults who carry excess weight* and* exhibit muscle weakness have a synergistic increased risk of functional impairment and subsequent disability than those who exhibit either symptom alone [10, 11].

Physical activity (PA) is associated with decreased risk of disability, delayed onset of functional impairment [12, 13], and recovery of function in older adults [14]. Physical activity is also associated with performance on key measures of physical performance including grip strength [15, 16]. Our group has previously demonstrated that PA moderates the risk of physical and functional limitations in normal weight and obese older adults [4, 17, 18]. Although previous work has demonstrated the association between grip strength and disability in middle and older adults [19, 20], whether higher levels of PA coupled with greater grip strength provides added benefits against physical and functional limitations in older adults who are obese is unknown. The current study extends our previous work to investigate the independent and combined effects of grip strength and physical activity on physical and functional outcomes in older adults with central adiposity. We examined (a) prevalence of physical and functional limitations by grip strength, (b) whether having higher grip strength was associated with reduced odds of physical and functional limitations, and (c) whether grip strength combined with PA is associated with additional reductions in physical and functional limitations. Given the compounded risk of disability among obese elders who also exhibit muscle weakness, it is important to identify ways of improving physical and functional outcomes within this particularly vulnerable group.

## 2. Methods

### 2.1. Data Source

This current study is a secondary analysis of existing data obtained from the 2006 and 2008 waves of the enhanced face-to-face interview of the Health and Retirement Study (HRS). HRS is a nationally representative panel survey of community dwelling adults aged 50 and older conducted by the University of Michigan and supported by the National Institute on Aging. The initial HRS sample was drawn in 1992 from a multistage, clustered area probability design of households, which targeted individuals born between 1931 and 1941. Follow-up interviews and new cohort additions have occurred at regular intervals and have resulted in a nationally representative sample of American adults over the age of 50. A random one-half of the larger HRS sample was preselected to complete an enhanced face-to-face interview in 2006, which included physical and biomarker measurements. The second half of the HRS sample received the enhanced face-to-face interview in 2008. Inclusion of both 2006 and 2008 waves ensures that all HRS respondents who were eligible for and consented to the enhanced face-to-face interviews are included in the analyses. Additional descriptions of sampling procedures and study design are available online at http://hrsonline.isr.umich.edu/.

### 2.2. Sample

A total of 6,555 community dwelling adults aged 60 years and older with clinically elevated waist circumference (WC ≥ 88 cm for women; WC ≥ 102 cm for men) [21, 22] and handgrip strength measurements were included in the sample. After eliminating respondents missing handgrip (*n* = 163) physical activity (*n* = 4), a total of 6,388 (2,364 men; 4,024 women) remained for analyses. Respondents of all races/ethnicities were included in the sample. Characteristics of those missing grip strength are provided in the Appendix. The study was exempted from Institutional Review Board review at all respective institutions due to the deidentified nature of the data used.

### 2.3. Measures

#### 2.3.1. Muscle/Grip Strength

Participants' handgrip strength was measured using Smedley spring-type hand dynamometer (TTM®, Tokyo, Japan) in a standing position with their arm at their side at a 90-degree angle. Two measurements were taken with each hand (dominant and nondominant) and averaged across trials [23]. The highest averaged score from either hand was used as primary predictor in the current analyses. Sex-specific tertiles were determined using univariate analyses. Participants were then classified into three categories by sex-specific grip tertiles: low grip (LG), mid grip (MG), and high grp (HG).

#### 2.3.2. Physical Activity

Physical activity (PA) was assessed using a self-report physical health questionnaire as part of the HRS interview. Respondents were asked* “How often do you take part in sports or activities that are vigorous, such as running or jogging, swimming, cycling, aerobics or gym, workout, tennis, or digging with a spade or shovel: more than once a week, once a week, one to three times a month, or hardly ever or never?”* and “*And how often do you take part in sports or activities that are moderately energetic such as, gardening, cleaning the car, walking at a moderate pace, dancing, floor or stretching exercises*…?” Frequency of engagement in HRS was reported as follows: more than once a week, once a week, one to three times a month, or hardly ever or never. Respondents reporting that they engaged in moderate or vigorous PA at least once per week were classified as active; those reporting activity less than once per week were classified as inactive (ref = 0).

#### 2.3.3. Physical Limitations (PL)

Physical limitations (PL) in HRS were assessed using a self-reported questionnaire. Respondents were asked to report whether they had difficulties* or* were unable to (yes/no) perform the following tasks because of a health or physical problem: walking several blocks, walking 1 block, sitting 2 hours, getting up from chair, climbing one flight of stairs, stooping, reaching arms, pulling/pushing large objects, lifting weights, and picking up a dime. All* yes* responses were compiled into a summary score in the HRS database ranging from 0 to 10. Univariate analyses revealed that 67% of our sample reported at least 1 limitation. For purposes of these analyses, respondents reporting difficulty or inability with performing two or more of the above tasks were classified as having physical limitations (0 = no limitations, 1 = limitation).

#### 2.3.4. Functional Limitations

Activities of daily living (ADLs) and instrumental activities of daily living (IADLs) were used to assess functional limitations [24]. Respondents were classified as having ADL limitations if they reported difficulty* or* inability with one or more of the following: dressing, eating, or getting out of bed. Respondents were classified as having IADL limitations if they reported difficulty or inability with at least one of the following IADLs: preparing meals, managing money, or needing help with house/yard work. All limitations reported in this paper were measured in HRS using self-report. Respondents were instructed to exclude any difficulties that were expected to last less than three months.

## 3. Analyses

Overall prevalence and odds of physical, ADL, and IADL limitations was examined by grip strength category. Prevalence and odds of limitations were also examined within each grip category by activity level. Multivariate logistic were run using PROC SURVEYLOGISTIC (SAS Institute Cary, NC). Models were adjusted for age (at time of physical assessment), years of education, ethnicity (white, black, and other), sex, smoking status (yes, no), body mass index (bmi) kg/m^2^, and number of medical comorbidities and weighted using the HRS respondent-level weights for physical measures, which includes adjustments for sample selection probability and nonresponse. Detailed documentation regarding HRS sample weight calculations is available at http://hrsonline.isr.umich.edu/sitedocs/wghtdoc.pdf. All data was examined using statistical tests that were two-sided with *p* values less than or equal to 0.05. Confidence intervals excluding 1.0 were considered statistically significant.

## 4. Results

Sample characteristics are presented in Table 1. The sample was predominantly white (83.4%) and female (63%). Overall prevalence of PL, ADL, and IADL limitations were 3,803 (59.5%), 1217 (28.2%), and 2404 (38.5%), respectively. Prevalence of PL, ADL, and IADL limitations was the highest in the lowest grip strength category, followed by MG, and was the lowest in the HG category. Prevalence of PL, ADL, and IADL limitations was also lower for those reporting engagement in physical activity within each grip category (Table 2).

Table 3 presents adjusted odds of PL, ADL, and IADL limitations by overall grip strength category and by activity level within each grip category. Overall odds of PL, ADL, and IADL limitation were significantly lower for those in MG category and the lowest in the HG category as compared to those in the LG grip category. In addition to increasing age, being female OR 2.00 [1.66, 2.42], *p* ≤ .001, BMI 1.08 [1.06–1.11], *p* ≤ .001, number of comorbidities 1.71 [1.57–1.85], *p* < .001, and being a current smoker 1.80 [1.30–2.48], *p* ≤ .01, were independently associated with higher odds of PL. These risk factors were also associated with increased odds of IADL limitations: OR 1.80 [1.49–2.19], *p* < .001 for female status, OR 1.04 [1.02–1.06], *p* ≤ .001 for BMI, OR 1.72 [1.58–1.87], *p* < .001 number of comorbidities, and OR 1.95 [1.42–2.67], *p* ≤ .001, for current smokers. For ADL limitations, race OR 1.39 [1.08, 1.78], *p* < .01, higher BMI OR 1.04 [1.02, 1.07], *p* < .01, and comorbidities OR 1.38 [1.26, 1.52], *p* ≤ .001, were associated with higher odds of limitation. Within grip categories, participating in physical activity did not appear to confer added benefits to reducing odds of disability or functional limitations.

## 5. Discussion

The current study examined the association between varying degrees of muscle strength, and odds of physical and functional limitations in older adults with central obesity who are physically active and inactive. We were primarily interested in (a) examining the prevalence of limitations by grip strength, (b) determining whether having higher grip strength was associated with reduced odds of physical and functional limitations, and (c) whether grip strength combined with PA is associated with additional reductions in physical and functional limitations. We found higher grip strength to be associated with lower prevalence of physical, ADL, and IADL limitations in elders with central obesity and increasing grip strength category to be significantly associated with progressively lower odds of physical, ADL, and IADL limitations after controlling for age, race, education, sex, BMI, medical comorbidities, and smoking status. This suggests that muscle strength is an important factor in preserving physical and functional impairment among those with central obesity independent of other known risk factors. Within grip categories, participating in physical activity was not associated with additional protection against odds of physical or functional limitations. In line with previous reports on risk factors for functional decline [25], we also found strong, independent associations between comorbidity and functional outcomes. These findings corroborate the need for targeted treatment and early intervention of chronic diseases among older adults, particularly the obese. Our findings extend the literature in that it provides supportive evidence to suggest that interventions aimed at improving muscle quality are beneficial even among obese elders with multiple health conditions.

Primary strengths of the current study include analyses of data from a nationally representative community dwelling sample of older adults with objective measures of grip strength and central adiposity (primary predictors). Limitations of the current analyses lie in the potential bias of self-reporting of physical outcomes and physical activity. In addition, the cross-sectional nature of the data limits our ability to make causal inferences regarding the association between muscle strength, physical activity, and functional outcomes in elders with central adiposity. We also cannot speak to the long-term impact of muscle strength on physical and functional outcomes in obese older adults. Additional research is needed to determine whether muscle strength and physical activity are protective against long-term functional decline in older adults with central obesity in the presence of other chronic health conditions.

## 6. Conclusion

Our results provide evidence that muscle strength is important for reducing odds of physical and functional limitations in obese elders. Among obese elders who are unable to engage in traditional aerobic activity, interventions that focus on preserving muscle strength are a viable means of reducing physical and functional limitations.

## Figures and Tables

**Table 1:** Sample characteristics.

*N*	6,388
	M (SD)

Age, years	71.4 (7.48)
Education, years	12.2 (3.13)
Comorbidities	2.2 (1.21)
Waist circumference (cm)	108.0 (12.50)
Handgrip strength	28.6 (10.43)
Body mass index (kg/m)^a^	29.9 (5.14)
Sex, *N* (%)	
Female	4,024 (63.0)
Race, *N* (%)^b^	
White	5,325 (83.4)
Black	866 (13.6)
Other	1,96 (3.1)
Current smoker, *N* (%)	636 (10.0)
Physically active, *N* (%)	3,493 (54.7)
Handgrip tertile^b^ *N* (%)	
Low	1,473 (23.1)
Mid	3,149 (49.3)
High	1,766 (27.7)
Physical limitations (PL)	3,803 (59.5)
Activities of daily living (ADL)	1,217 (28.2)
Instrumental activities of daily living (IADL)	2,404 (38.5)

^a^ *N* = 6,279 with BMI measurements.

^b^Low grip ≤ 31.25 men, ≤ 18.5 women; high grip ≥ 43.5 men, ≥ 26.5 women.

**Table 2:** Prevalence of physical, ADL and IADL limitations by grip tertiles and physical activity.

	Low grip			Mid grip			High grip		
	Overall	Low PA	High PA	Overall	Low PA	High PA	Overall	Low PA	High PA
Physical	1126 (76.4)	698 (84.8)	428 (65.9)	1839 (58.4)	1002 (70.9)	837 (48.2)	838 (47.5)	395 (59.9)	443 (40.0)
ADL	438 (37.4)	321 (45.6)	117 (25.1)	547 (25.6)	337 (30.7)	210 (20.1)	232 (23.0)	132 (28.2)	100 (18.5)
IADL	826 (57.6)	556 (69.3)	270 (42.8)	1141 (37.1)	689 (49.6)	452 (26.7)	437 (25.2)	237 (36.1)	200 (18.6)

*Note*. Low grip ≤ 31.25 men, ≤ 18.5 women; high grip ≥ 43.5 men, ≥ 26.5 women. ADL limitations difficulty or inability with ≥1 dressing, eating, or getting out of bed. IADL ≥1 preparing meals, managing money, house/yard work.

**Table 3:** Odds of physical and ADL limitations by grip tertile and activity level.

	*N*	Mid grip^ab^			High grip^ab^		
		Overall	Low activity	High activity	Overall	Low activity	High activity
Physical	6,388	0.54 (0.42, 0.69)	0.62 (0.42, 0.92)	0.54 (0.39, 0.75)	0.42 (0.31, 0.56)	0.40 (0.25, 0.63)	0.48 (0.33, 0.70)
ADL	4,320	0.58 (0.45, 0.76)	0.45 (0.32, 0.64)	0.91 (0.61, 1.37)	0.60 (0.43, 0.84)	0.50 (0.32, 0.79)	0.87 (0.53, 1.46)
IADL	6,248	0.59 (0.47, 0.75)	0.58 (0.41, 0.81)	0.69 (0.49, 0.97)	0.46 (0.34, 0.61)	0.40 (0.26, 0.61)	0.61 (0.40, 0.92)

Referent: low grip. ADL: activities of daily living, IADL: instrumental activities of daily living.

^a^Models are weighted and adjusted for age, sex, education, race, number of comorbidities, current smoking status, and body mass index.

^b^Observations with zero or negative weights do not contribute to estimates and were automatically eliminated from the analyses (*n* = 3,028).

**Table 4:** Sample characteristics of missing grip.

*N*	163
Age, years	72.2 (7.80)
Education, years	11.22 (3.36)
Comorbidities	2.72 (1.23)
Waist circumference (cm)	108.0 (14.19)
Body mass index (kg/m)	29.9 (5.01)
Sex, *N* (%)	
Female	142 (89.0)
Race, *N* (%)	
White	115 (70.55)
Black	36 (22.09)
Other	12 (7.36)
Current smoker, *N* (%)	20 (12.27)
Physically active, *N* (%)	56 (34.366)

## Appendix

See Table 4.
